# Effects of teicoplanin on cell number of cultured cell lines

**DOI:** 10.1515/intox-2015-0004

**Published:** 2015-03

**Authors:** Tahere Kashkolinejad-Koohi, Iraj Saadat, Mostafa Saadat

**Affiliations:** 1 Department of Biology, College of Sciences, Shiraz University, Shiraz, Iran; 2 Institute of Biotechnology, Shiraz University, Shiraz, Iran

**Keywords:** human cell lines, MTT, proliferation, teicoplanin, toxicology

## Abstract

Teicoplanin is a glycopeptide antibiotic with a wide variation in human serum half-life. It is also a valuable alternative of vancomycin. There is however no study on its effect on cultured cells. The aim of the present study was to test the effect of teicoplanin on cultured cell lines CHO, Jurkat E6.1 and MCF-7. The cultured cells were exposed to teicoplanin at final concentrations of 0–11000 μg/ml for 24 hours. To determine cell viability, the 3-(4,5-dimethylthiazol-2-yl)-2,5-diphenyltetrazolium bromide (MTT) test was performed. At low concentrations of teicoplanin the numbers of cultured cells (due to cell proliferation) were increased in the three cell lines examined. The maximum cell proliferation rates were observed at concentrations of 1000, 400, and 200 μg/ml of teicoplanin for CHO, MCF-7 and Jurkat cell lines, respectively. Cell toxicity was observed at final concentrations over 2000, 6000, and 400 μg/ml of teicoplanin for CHO, MCF-7 and Jurkat cell lines, respectively. A dose-dependent manner of cell toxicity was observed. Our present findings indicated that teicoplanin at clinically used concentrations induced cell proliferation. It should therefore be used cautiously, particularly in children, pregnant women and patients with cancer.

## Introduction

Teicoplanin (extracted from *Actinoplanes teichomyceticus)* is an antibiotic used in the prophylaxis and treatment of serious infections caused by Gram-positive bacteria, including methicillin-resistant *Staphylococcus aureus* and *Enterococcus faecalis.* The molecular structure of teicoplanin is related to that of vancomycin with a similar spectrum of activity (Eggleston and Ofosu 1988). Its mechanism of action is to inhibit peptidoglycan polymerization, resulting in inhibition of synthesis of Gram-positive bacteria cell walls and consequent cell death (Somma *et al*., 1984; Jovetic *et al*., 2010). It is marketed under the trade name Targocid. During the last decade, an ever-increasing number or clinical studies has been performed, covering a large spectrum of clinical indications in various groups of patients (Glupezynski *et al*., 1986). Teicoplanin is predominantly bound to plasma proteins. It is not absorbed orally, but intravenous and intramuscular administration is well tolerated. Teicoplanin is eliminated predominantly by the kidneys and only 2 to 3% of an intravenously administered dose is metabolized.

Due to its low rate of side effects (Bibler *et al*., 1987; Matthews *et al*., 2014), thus not requiring close monitoring, and its longer serum half-life (Outman *et al*., 1990; Tobin *et al*., 2010) teicoplanin is a valuable alternative to vancomycin and has become the glycopeptide of choice in many hospitals (Glupezynski *et al*., 1986; Guzek *et al*., 2013; Salimi *et al*., 2014).

From the clinical point of view, the potentially antibacterial effect of teicoplanin was subjected to comprehensive investigation of its antibacterial effect and minimal cytotoxic properties in eukaryotic cells. The aim of the present study was to test the ability of teicoplanin to induce cytotoxic effects on cultured cell lines.

## Materials and methods

CHO, Jurkat E6.1 and MCF-7 cell lines were used in the present study. The cell lines were cultured at 37 °C in a humidified 5% CO_2_ atmosphere in plastic dishes in RPMI-1640 medium supplemented with 10% heat-inactivated fetal calf serum, 2 mM L-glutamine, and antibiotics (100 units/ml penicillin and 100 μg/ml streptomycin).

Teicoplanin at final concentrations of 0–11000 μg/ml was added to 500 μl of the cultured cell lines onto plates. The treated cells were incubated for 24 hours at 37 °C in 5% CO_2_. After treatment, the viability of the cells was evaluated by the 3-(4,5-dimethylthiazol-2-yl)-2,5-diphenyltetrazolium bromide (MTT) assay. MTT reagent was added to each plate and after 6 h of incubation 1ml of SDS (10% in 0.01N HCl) was added to dissolve the water-insoluble formazan salt. OD570 nm was measured. Unexposed cells were regarded as 100% viable.

All the values in this study are expressed as the mean ± SD of three independent experiments.

## Results and discussion

**Figure 1:**
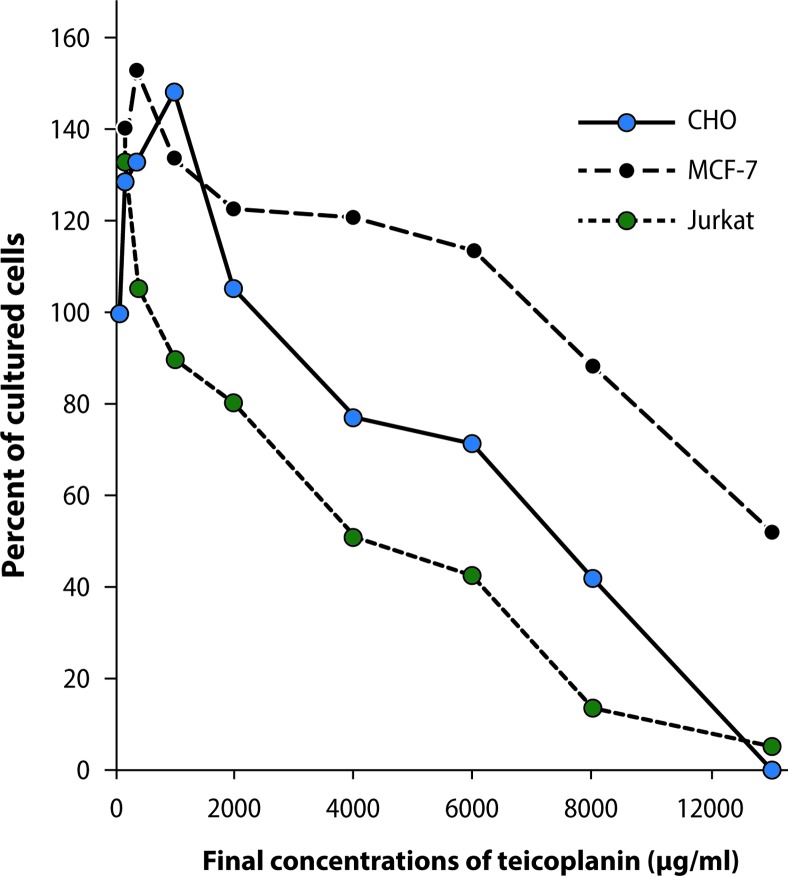
Effects of teicoplanin on cultured CHO, MCF-7 and Jurkat cell lines.

Figure 1 shows the percentage of viability of the cell lines studied after 24-hour treatment with teicoplanin at concentrations ranging from 0 to 11000 μg/ml, measured by the MTT assay. Our findings indicated two different effects of teicoplanin on proliferation of the cell lines examined. At low concentrations of teicoplanin, the numbers of cultured cells (due to cell proliferation) were increased in the three cell lines studied. The maximum cell proliferation rates were observed at teicoplanin concentrations of 1000, 400, and 200 μg/ml for CHO, MCF-7 and Jurkat cell lines, respectively. Teicoplanin induced cell proliferation up to final concentrations of 2000, 6000, and 400 μg/ml for CHO, MCF-7 and Jurkat cell lines, respectively. After these concentrations, a dose-dependent manner of cell toxicity was observed. At the final concentration of 11000 μg/ml of teicoplanin, only 0.3, 52.4, and 5.2 percent of the respective CHO, MCF-7 and Jurkat cells exhibited viability. The experiments were carried out for CHO cells after 48 and 72 hours of incubation. The same results were observed.

On balance, teicoplanin exerted dual effects on the cell lines examined. First it induced cell proliferation and then it showed cell toxicity. It should be mentioned that in light of our data cell proliferation and cell toxicity effects of teicoplanin showed cell specific patterns.

The concentrations of teicoplanin which revealed cell toxicity were much higher than the clinically used concentrations (Tobin *et al*., 2010; Matthews *et al*., 2014; Salimi *et al*., 2014; Oda *et al*., 2014). It might thus be concluded that teicoplanin appears to be a safe drug. Toxicological studies have shown that teicoplanin does not cause toxicity, compared with vancomycin (Verbist *et al*., 1984; Bibler *et al*., 1987; Matthews *et al*., 2014). Based on our present findings, it should be noted that teicoplanin at clinically used concentrations induced cell proliferations. We are not sure whether it is safe or if it is possibly associated with pathological cell growth (malignant cells and/or initiate cells).

Finally it should be mentioned that teicoplanin is to be used with caution particularly in children, pregnant women and patients with cancer. Further experiments are necessary to clarify the significance of the present findings and the cancer risk associated with the use of teicoplanin.
